# Selective Lymphatic Duct Embolization in Patients With Chylothorax and Thoracic Duct Side-Branch Injury

**DOI:** 10.7759/cureus.84754

**Published:** 2025-05-24

**Authors:** Sami Soliman, Joe Khoury, Hakob Kocharyan, Christopher Yeisley, Elias Salloum, Mustafa Al-Roubaie

**Affiliations:** 1 College of Medicine, University of South Florida Morsani College of Medicine, Tampa, USA; 2 Interventional Radiology, Emory University School of Medicine, Atlanta, USA; 3 Interventional Radiology, Moffitt Cancer Center, Tampa, USA; 4 Interventional Radiology, Naval Medical Center Portsmouth, Portsmouth, USA

**Keywords:** chylothorax, interventional radiology, lymphangiography, postoperative management, selective thoracic duct embolization, thoracic duct embolization

## Abstract

Chylothorax, the accumulation of chyle in the pleural space, is a potentially fatal condition that necessitates intervention. Options range from conservative treatment, such as dietary modifications and pharmacological therapy, to percutaneous and surgical interventions. This case describes a 56-year-old male status post-lung wedge resection who developed a high-output chylothorax. Initial management with octreotide and a low-fat diet was unsuccessful. A percutaneous lymphangiogram identified extravasation of contrast from a thoracic duct side-branch. Instead of traditional thoracic duct embolization (TDE), selective lymphatic duct embolization (SLDE) was performed to preserve the main thoracic duct. The patient had rapid symptom resolution and successful chest tube removal. The patient was discharged within two days, without recurrence of symptoms. While TDE is a common percutaneous approach, it carries the risk of complete thoracic duct occlusion. This can lead to lymphatic backflow and leaks. When a single branch of the thoracic duct is injured, SLDE offers a novel alternative to target that side-branch while maintaining thoracic duct integrity. Comparative studies suggest SLDE provides similar efficacy to TDE while reducing severe complications. More research is needed to establish the long-term risks versus benefits of SLDE. SLDE is a promising, minimally invasive intervention for high-output chylothorax. It offers a safer alternative to TDE when a side-branch injury exists. This case highlights its potential for improving patient outcomes with fewer complications.

## Introduction

Chylothorax is the accumulation of chyle in the pleural space, often due to traumatic (iatrogenic or non-iatrogenic), nontraumatic, or idiopathic etiologies [1]. Among those of traumatic origins, iatrogenic or surgical causes account for 25%-50% of cases, including those secondary to abdominal surgery or pacemaker placement [1]. Nontraumatic causes account for 25%-50% of cases and include lymphatic disorders, malignancy-related causes, and alterations in lymph composition or volume [1]. Idiopathic etiologies account for ~6% of the causes of chylothoraces [1]. Symptoms of dyspnea, a sensation of fullness, and pleuritic chest pain often require frequent drainages or prolonged chest tube placement. Treatments of chylothorax depend on the etiology and severity of the disease. Some conservative measures include dietary modification or even complete parenteral nutrition in some severe post-surgical cases. Conservative measures may be effective in 20%-80% of cases, depending on the scenario, but many patients with high-volume chylothorax will require further surgical or interventional treatments [1]. Surgical management may include complete ligation of the thoracic duct or repair of pleural defects, which will prevent leakage into the thorax from a lymphatic injury in the chest. Interventional Radiology can assess and treat lymphatic injury by accessing the thoracic duct with a retrograde venous approach at the junction of the left internal jugular vein and subclavian veins. Percutaneous thoracic duct access can also be obtained following lymphangiography with lipiodol by puncturing the cisterna chyli with a percutaneous needle. Once thoracic duct access is achieved, complete thoracic duct embolization (TDE) is often performed. In rare cases of a specifically injured thoracic duct side-branch, selective embolization may be used to preserve the patency of the main thoracic duct. Here, we present a case of a patient who developed a high-output chylothorax post-lung surgery, successfully managed through selective lymphatic duct embolization (SLDE) of an injured thoracic duct side-branch, thus preserving the integrity of the main thoracic duct and minimizing associated complications.

## Case presentation

A 56-year-old male with a history of smoking was incidentally found to have a 2.7 cm right upper lobe lung nodule on a chest CT that was obtained after a motor vehicle collision. He was discharged from the emergency department with self-limited injuries and was referred to a regional cancer center for lung nodule management. He subsequently underwent an endobronchial ultrasound-guided biopsy by an interventional pulmonologist, which resulted in a diagnosis of adenocarcinoma of the lung. A PET/CT confirmed no other sites of disease. After cardiac clearance was confirmed by cardiology, including an unremarkable echocardiogram, he then underwent a video-assisted wedge resection and surgical chest tube placement with thoracic surgery. The procedure was uneventful; however, on postoperative day 2, his chest tube output increased to over 1 L per day of chylous fluid. The patient reported fatigue and mild respiratory distress with walking. Conservative management with octreotide 200 µg three times daily and a low-fat diet was initiated, but the patient showed minimal improvement after five days.

**Figure 1:**
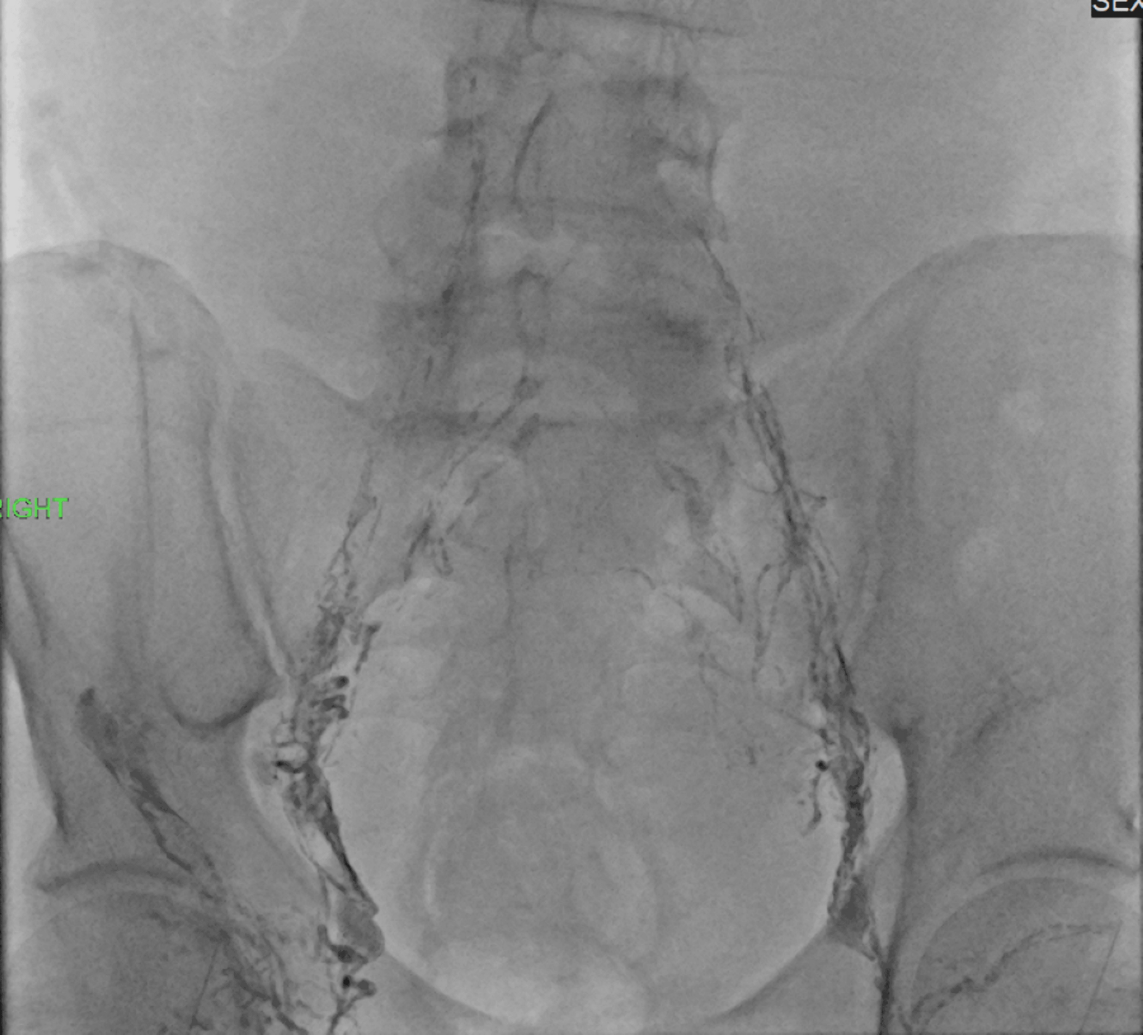
After ultrasound-guided puncture of several bilateral inguinal lymph nodes with 25G needles, lipiodol was injected, and pelvic lymphangiography was obtained

**Figure 2:**
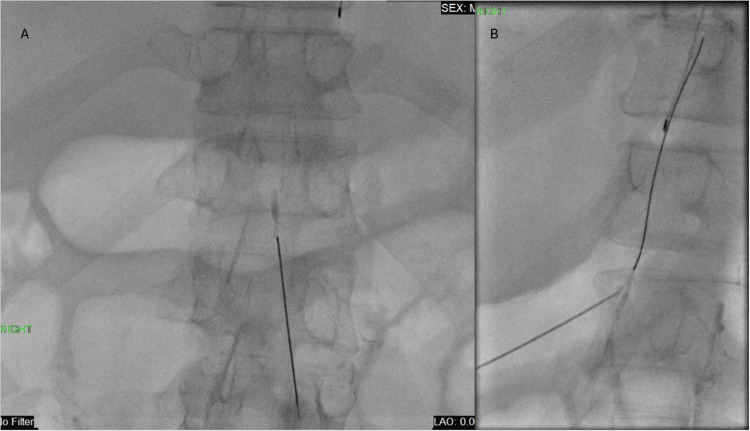
Fluoroscopic spot film demonstrates percutaneous access to the cisterna chyli with a 22G needle (A) and subsequent threading of a 0.018-inch guidewire into the thoracic duct (B)

**Figure 3:**
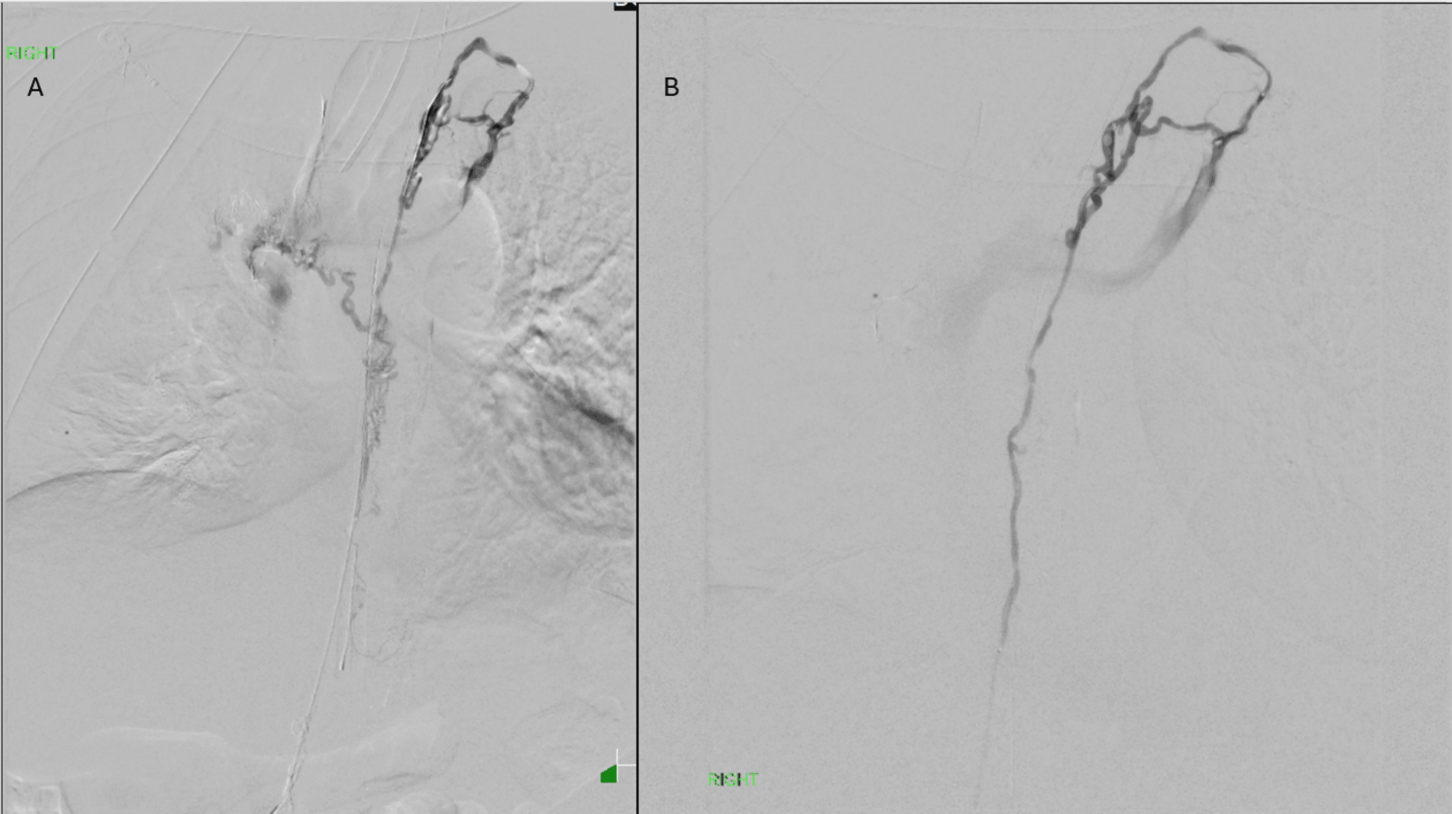
Thoracic duct lymphangiography through a 2.4F microcatheter demonstrates extravasation of contrast into the right hemithorax surgical bed (A). The site of injury was localized to a right-side branch of the main thoracic duct. After careful superselection of the injured branch, embolization of the focal injury was performed with an aliquot of 0.1 mL of 3:1 glue, followed by D5W to clear the microcatheter and preserve access into the thoracic duct to allow for post-embolization lymphangiography, as opposed to the typical withdrawal of the microcatheter after glue embolization. Post-embolization lymphangiography demonstrated no further extravasation, a patent main thoracic duct, and normal filling into the systemic venous system (B)

Given the high output of the chest tube and minimal improvement with medical management, the decision was made to forgo further conservative measures, such as initiating total parenteral nutrition with strict nil per os, and instead to proceed with interventional management. A percutaneous lymphangiogram was performed by an interventional radiologist in the angiography suite. General anesthesia was provided by an anesthesiologist. Under ultrasound guidance, several bilateral inguinal lymph nodes were accessed using 25G needles at the cortex-hilum interface, and lipiodol was manually injected slowly, producing a pelvic lymphangiogram (Figure 1). Venous compression devices were applied to the patient’s calves bilaterally to facilitate lipiodol ascension up the lymphatic chain to the cisterna chyli. As the lipiodol ascended the lymphatic system to the cisterna chyli, a 22G needle was used to puncture the cisterna chyli transabdominal above the navel using a fluoroscopically guided technique (Figure 2A), and a 0.018-inch wire was advanced into the thoracic duct (Figure 2B). Over the wire, the needle was exchanged for a 2.0 French microcatheter, through which contrast was injected, and a thoracic duct lymphangiography was obtained. Breathholds facilitated by the anesthesiologist significantly reduced motion artifacts during the thoracic lymphangiograms. The findings demonstrated extravasation of contrast into the right hemithorax surgical bed via a side-branch of the main thoracic duct (Figure 3A). There was a brisk flow of contrast out of the injured side-branch. The main thoracic duct was intact and served as the primary conduit of lymphatic flow into the left subclavian vein. Given the precise identification of the injured side-branch causing the chyle leak and the importance of keeping the main thoracic duct intact, the decision was made to attempt super-selection and embolization of the injured side-branch. The microcatheter, along with a 0.014-inch microwire, was then guided into the injured side-branch under fluoroscopic guidance. Superselective embolization of the injured side-branch was performed with a gentle 0.1 mL aliquot of lipiodol and glue (3:1 mixture, respectively) to avoid reflux and non-target embolization. Glue was used as opposed to microcoils due to tenuous superselective access into the lymphatic side-branch. The use of microcoils may have inadvertently dislodged the microcatheter from the selected side-branch during deployment. Post-embolization lymphangiography demonstrated resolution of the extravasation while maintaining an intact main thoracic duct (Figure 3B). The microcatheter and bilateral groin needles were removed and hemostasis was achieved with manual pressure. The total procedure time was three hours and 38 minutes, the radiation time was 48 minutes, and the total radiation dose was 289 mGy. The patient was monitored in the recovery room for two hours and was discharged to the floor hemodynamically stable and without respiratory distress.

The following day, the patient had no chest tube output over 24 hours, and a chest X-ray demonstrated no pleural effusion. Two days post-procedure, the chest tube was removed, and the patient was discharged home. The final pathology of the resected lung specimen confirmed complete excision with negative margins. The patient followed up in the IR clinic after two weeks with no evidence of post-procedural complications. Chest X-ray demonstrated no re-accumulation of effusion. The patient denied respiratory distress, abdominal swelling, lower extremity swelling, or diarrhea.

## Discussion

Appropriate management of a chylothorax, independent of etiology, is important. Conservative management can include dietary modifications to reduce chyle production and facilitate healing, as well as pharmacologic interventions such as somatostatin, etilefrine, or nitric oxide, which may also be beneficial [2,3]. Conservative management is successful in 50% of chylothoraces of non-malignant etiologies but less so in those of neoplastic origin [4]. Despite a chylothorax of non-malignant origin in our patient, conservative treatment was not effective, warranting further intervention.

Surgical options including thoracic duct ligation, pleurodesis, and pleuroperitoneal shunting were considered. They offer greater success compared to conservative management but necessitate good surgical candidacy and can be associated with mortality as high as 25% and complications as high as 38% [1]. Meanwhile, percutaneous interventions have a complication rate of ~3% [1]. This patient required a confirmatory diagnosis and localization of the leak, so a percutaneous lymphangiogram was conducted. This afforded the opportunity for immediate repair via a traditional approach such as TDE. This technique, while less successful than surgery, is better tolerated by patients [2]. The most common complications of TDE by percutaneous embolization or surgical ligation include lower extremity lymphedema, chylous ascites, chronic diarrhea, and malnutrition due to protein-losing enteropathy [5]. These complications occur due to the complete blockage of the thoracic duct; therefore, superselective embolization of a thoracic duct side-branch, which maintains patency of the main thoracic duct, greatly reduces the chances of these complications. The novel use of SLDE in this case highlights comparable efficacy and reduced short and long-term complications compared to TDE. Similar results have been found in a study conducted by Srinivasan et al., where TDE vs. SLDE was used in children with thoracic lymphatic flow disorders. They found that 85% of patients who underwent SLDE, compared to 72% of those who received TDE, experienced symptom resolution (P=0.001). SLDE was associated with a higher incidence of adverse events (14/39 vs. 17/104), including mild post-op hypotension, SIRS, and reflux of glue, compared to TDE; however, more severe complications, such as cardiac arrest and intra-abdominal hemorrhage, were observed in the latter [6]. Of note, no statistical testing was conducted to compare the incidence of the listed complications [6]. This study also did not assess long-term outcomes, but a 2012 study reported a 14.3% incidence of likely long-term complications following TDE [5]. Additionally, Arslan et al. showed that superselective retrograde lymphatic duct embolization can be a feasible alternative for the management of a postoperative lymphatic leak, as it allows for targeted embolization of the affected branch and may reduce complications associated with traditional TDE [7].

Although percutaneous fluoroscopic lymphangiography is often utilized for both diagnosis and treatment, it is important to note that dynamic contrast-enhanced (DCE) magnetic resonance (MR) lymphangiography and nuclear medicine (NM) lymphangiogram are alternative diagnostic tools for evaluating patients with suspected thoracic duct injury. DCE MR lymphangiography is radiation-free and offers high spatial resolution, which can help detect the location of thoracic duct injury for either surgical planning or even SLDE in select patients [8]. NM lymphangiogram can be performed with a low radiation dose and provide real-time imaging of lymphatic flow [9].

## Conclusions

Chylothorax remains a life-threatening condition that requires timely diagnosis and management. This case and emerging data show that SLDE offers a promising alternative to TDE in cases of lymphatic side-branch injury. SLDE, compared to TDE, offers comparable efficacy and possibly fewer long-term complications due to the preservation of the main thoracic duct. Although SLDE may be more technically demanding than conventional approaches, it is a more refined and tailored solution that justifies its complexity. Further prospective studies are warranted to further evaluate the long-term outcomes and safety of SLDE.
